# Regulatory Mechanism on Anti-Glycolytic and Anti-Metastatic Activities Induced by *Strobilanthes crispus* in Breast Cancer, In Vitro

**DOI:** 10.3390/ph16020153

**Published:** 2023-01-20

**Authors:** Siti Nur Hasyila Muhammad, Nur Arnida Mohd Safuwan, Nik Soriani Yaacob, Agustine Nengsih Fauzi

**Affiliations:** Department of Chemical Pathology, School of Medical Sciences, Universiti Sains Malaysia, Kubang Kerian 16150, Kelantan, Malaysia

**Keywords:** *Strobilanthes crispus*, breast cancer, anti-glycolytic, anti-metastatic, MDA-MB-231

## Abstract

An active fraction of *S. crispus*, F3, and its bioactive compounds (lutein, β-sitosterol, and stigmasterol) were reported to have anti-glycolytic activities in MDA-MB-231 cells. Since glycolysis can also regulate metastatic activities in cancer cells, this study investigated the mechanism underlying the anti-glycolytic and anti-metastatic activities induced by F3 and its bioactive compounds on MDA-MB-231 cells. The cells were treated with IC_50_ concentrations of F3, lutein, β-sitosterol, and stigmasterol. GLUT1 protein expression and localization were then observed using a fluorescence microscope. We found that F3, lutein, and β-sitosterol inhibit localization of GLUT1 to the cell membrane, which causes the decrease in glucose uptake. This is supported by a reduction in PKC activity, measured using a spectrophotometer, and increased TXNIP protein expression detected by Western blotting. Both TXNIP and PKC are involved in GLUT1 activation and localization. The expression of signaling proteins involved in the PI3K/AKT pathway was also measured using a flow cytometer. Results show that F3, lutein, β-sitosterol, and stigmasterol reduced the expression of AKT, pAKT, mTOR, and HIF1α in MDA-MB-231 cells. Transwell migration assay was used to measure migration of the MDA-MB-231 cells. A reduction in fibronectin protein expression was observed by fluorescence microscopy, after treatments with F3 and its bioactive compounds, leading to a reduction in the MDA-MB-231 cells’ migratory abilities. As a conclusion, F3 acts as a metabolic inhibitor by inhibiting metabolic rewiring in the promotion of cancer metastasis, potentially due to the presence of its bioactive compounds.

## 1. Introduction

Cancer is a complex disease, with increased glucose uptake and lactate production even under aerobic conditions [1]. This occurs via aerobic glycolysis, also known as the Warburg effect [2], in which the main function is to provide rapid energy production to support highly proliferated cancer cells [3]. Evidence shows that tumor initiation and progression require increased production of ATP and macromolecules by strongly relying on the reprogramming of cell metabolism [4]. Furthermore, alteration of metabolic enzymes such as hexokinase 2 (HK2), pyruvate kinase type M2 (PKM2), and lactate dehydrogenase (LDH), as well as glucose transporters (GLUTs) and lactate transporters (monocarboxylate transporters, MCTs), have been observed in the Warburg effect [5,6,7,8]. Therefore, targeting tumor cell metabolism to achieve therapeutic benefits in cancer treatments has garnered interest among researchers.

Besides enhancing cellular proliferation, the Warburg effect promotes cancer metastasis. During metastasis, malignant cells disseminate from their primary sites and grow in distant organs [9]. Restraining oxidative metabolism enables cancer cells to avoid excess reactive oxygen species (ROS) generation, thus, increasing their survival during metastasis [10]. Low ROS production can protect cancer cells from ROS-mediating anoikis, which is a form of cell death that occurs when the cells lose contact with their neighboring cells and the extracellular matrix [11,12]. Over 90% of breast cancer deaths are related to metastasis [13]. Multiple steps are known to be involved during metastasis: (1) Epithelial-mesenchymal transitions (EMT), whereby the epithelial cells lose their polarity and gain mesenchymal phenotypes causing them to become highly mobile and invasive. This invasiveness of the cells facilitates their migration from their primary site to the distal site [14]; (2) Tissue invasion through the degradation of the extracellular matrix (ECM); (3) Intravasation and penetration of the cancer cells to the blood vessel wall and entry into the circulation as circulating tumor cells (CTCs); (4) Homing and survival of the CTCs within the circulation; (5) The extravasation of cancer cells passes through the vascular wall and exits the bloodstream of the distant organ; (6) Formation of a metastatic niche via angiogenesis (formation of new blood vessels) [15]. 

The PI3K/AKT/mTOR signaling pathway is important in driving tumorigenesis and the progression of cancer cells. Conversely, activation of mTOR signaling has been reported to drive changes in cancer cell metabolism by promoting several metabolic pathways such as glucose, amino acid, nucleotide, fatty acid, and lipid metabolism [16]. During glucose metabolism, activation of mTOR signaling in cancer cells can enhance the expression of genes related to glucose uptake and glycolysis through upregulation of the hypoxia-inducible factor 1α (HIF1α) [17]. The function of this transcription factor is to regulate more than 1000 target genes that are important for the survival of cancer cells including proliferation, angiogenesis, EMT, invasion, metastasis, and metabolic reprogramming [18]. High expression of HIF1α has been related to poor prognosis in breast cancer patients [19,20]. One of the potent negative regulators for glucose uptake and aerobic glycolysis is the thioredoxin-interacting protein (TXNIP) [21]. TXNIP suppression has been shown to increase glucose metabolism in triple negative breast cancer cells (TNBC), thereby promoting the growth and survival of this aggressive breast cancer subtype [22].

**Figure 1:**
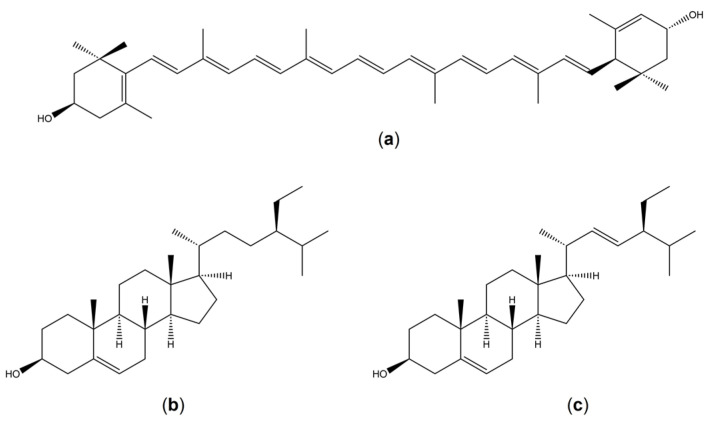
The chemical structures the bioactive compounds isolated from F3: (**a**) lutein; (**b**) β-sitosterol and (**c**) stigmasterol.

*Strobilanthes crispus* (L.) Blume (*S. crispus*) is a Malaysian medicinal plant that has been reported to have anticancer activity on several cancer cell lines [23,24,25], including caspase-dependent apoptosis and cell cycle arrest in human breast cancer cell lines with no effects on the non-malignant cells [25,26]. F3, a bioactive subfraction extracted from *S. crispus* leaves, can significantly cause tumor regression, and inhibit metastasis in *N*-Methyl-*N*-nitrosourea (NMU) induced rat tumor models [27,28]. High-performance liquid chromatography (HPLC) and nuclear magnetic resonance (NMR) identified six bioactive compounds in the F3 including lutein, β-sitosterol, and stigmasterol (Figure 1) [29]. Along with F3, lutein, and β-sitosterol also caused a reduction in tumor growth and prevented secondary tumor development in the 4T1-induced mouse mammary carcinoma model [30].

*S. crispus* is traditionally used to treat diabetes, and daily consumption of *S. crispus* tea has been shown to reduce blood glucose in hyperglycemic rats [31]. Since cancer cells depend on glucose for energy production, we examined the effects of F3 on the glucose metabolism of TNBC, MDA-MB-231. Interestingly, our latest findings showed that F3 inhibited glucose uptake and lactate production in breast cancer cells via its bioactive compounds [32]. The current study focused on the effects of F3 and its bioactive compounds on anti-glycolytic and anti-metastatic activities by investigating their effects on glucose transporter 1 (GLUT1) localization and the expression of glycolytic and metastatic regulatory molecules in the MDA-MB-231 cells. MDA-MB-231 cells are characterized by GLUT1 overexpression and high glucose consumption [33] activated by the PI3K/AKT/mTOR pathway.

## 2. Results

### 2.1. Effects of F3, Lutein, β-sitosterol, and Stigmasterol on GLUT1 Protein Expression and Localization

**Figure 2:**
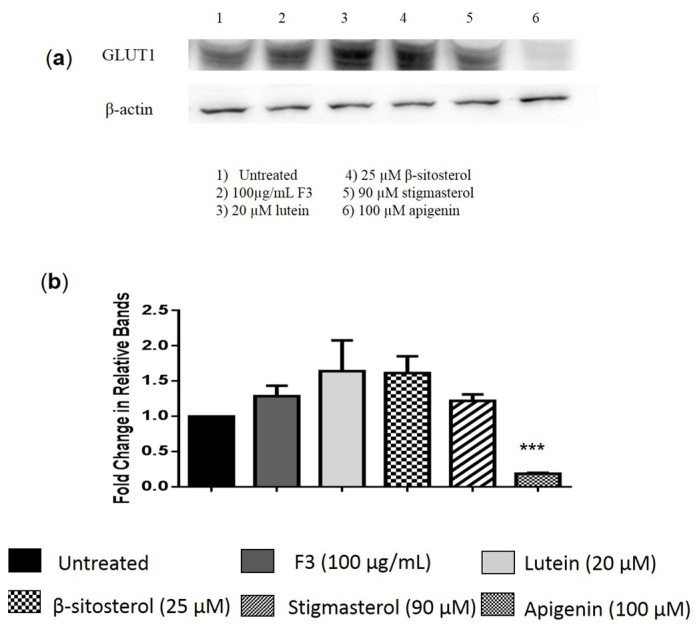
GLUT1 protein expression. (**a**) Representative image of Western blot analysis of MDA-MB-231 cells treated with the indicated concentrations of (2) F3, (3) lutein, (4) β-sitosterol, (5) stigmasterol, and (6) apigenin (positive control), while (1) untreated as a negative control for 24 h. (**b**) Results are presented as an average fold change in the level of each protein normalized to β-actin ± SD (*n* = 3). *** *p* < 0.001.

**Figure 3:**
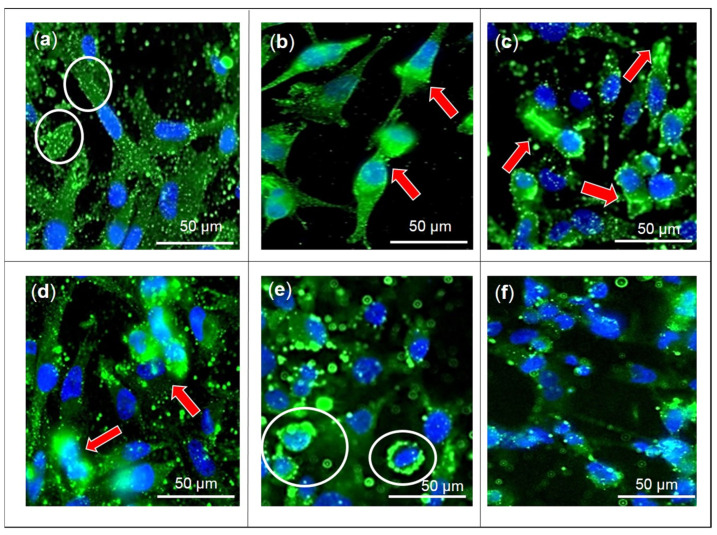
Localization of GLUT1 to the cell membrane. MDA-MB-231 cells were untreated (negative control; (**a**) or treated with the indicated concentrations of (**b**) F3, (**c**) lutein, (**d**) β-sitosterol, (**e**) stigmasterol, and (**f**) apigenin (positive control) for 24 h. GLUT1 expression and localization to the cell membrane was observed under a fluorescence microscope at 400× magnification. Red arrows show an abundance of green punctate in the nucleus and cytoplasm area of treated cells. White circles show GLUT1 localization at the plasma membrane.

GLUT1 protein expression in MDA-MB-231 cells was unaffected by F3 and its bioactive compounds after being treated for 24 h (Figure 2a and Figure S1). However, only apigenin-treated cells showed a significant reduction in GLUT1 protein expression in MDA-MB-231 cells (Figure 2b). Meanwhile, localization of GLUT1 to the cell membrane was observed using fluorescent microscopy (Figure 3). The fluorescent microscopy results show high green intensity prominently in the plasma membrane area of untreated cells, indicating localization of GLUT1 (Figure 3). For MDA-MB-231 cells treated with F3 (Figure 3b), lutein (Figure 3c), and β-sitosterol (Figure 3d) for 24 h, the green fluorescence of GLUT1 appears as punctate structures distributed in the cytoplasm and nuclear region of the cells compared to the control (Figure 3a) where more green fluorescence was observed in the plasma membrane area. MDA-MB-231 cells treated with stigmasterol (Figure 3e) show green intensity comparable to the control cells with less green punctate observed in the nucleus and cytoplasm area. 

### 2.2. Effects of F3, Lutein, β-sitosterol, and Stigmasterol on PKC Activity

**Figure 4:**
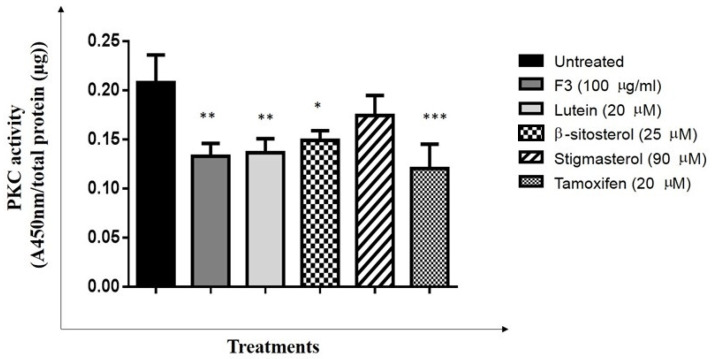
PKC activity. MDA-MB-231 cells were treated with IC_50_ concentrations of F3, lutein, β-sitosterol, and stigmasterol for 24 h. The PKC activity in treated cells was measured using a spectrophotometer. Data are presented as the mean ± SD (*n* = 3). * *p* < 0.05; ** *p* < 0.01; *** *p* < 0.001.

To elucidate the effect of F3 and its bioactive compounds on PKC activity, the cells were treated with F3, lutein, stigmasterol, and β-sitosterol for 24 h. The cell lysates were collected and assessed for PKC activity by in vitro kinase assay. The PKC activity was significantly reduced in all treated cells, except for stigmasterol, compared to the untreated control after 24 h incubation (Figure 4). 

### 2.3. Effects of F3, Lutein, β-sitosterol, and Stigmasterol on TXNIP and HK2 Protein Expressions

**Figure 5:**
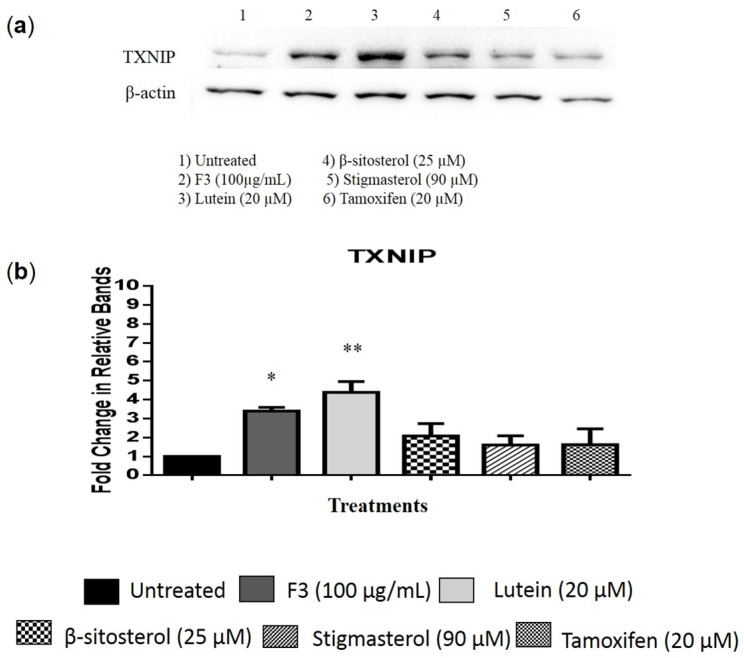
TXNIP protein expression. MDA-MB-231 cells were treated with the indicated concentrations of (2) F3, (3) lutein, (4) β-sitosterol, and (5) stigmasterol for 24 h. The protein expression was then determined by Western blotting. (**a**) Representative image of TXNIP protein expression. Tamoxifen was used as a positive control, while untreated cells were used as negative control. (**b**) Results are presented as an average fold change in the level of each protein normalized to β-actin ± SD (*n* = 3). * *p* < 0.05; ** *p* < 0.01.

**Figure 6:**
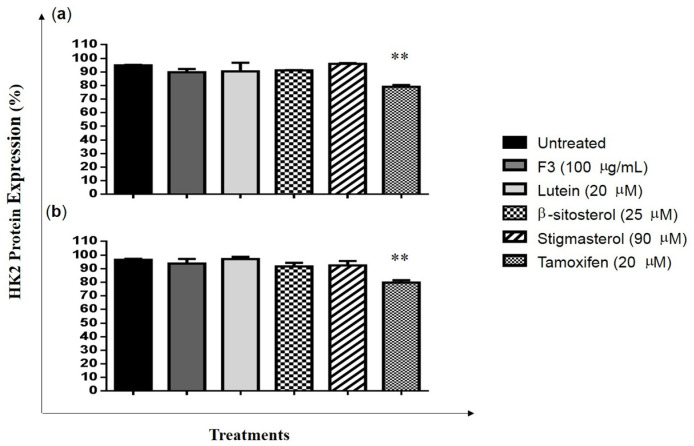
MDA-MB-231 cells expressed with HK2 protein. MDA-MB-231 cells were treated with IC_50_ concentrations of F3, lutein, β-sitosterol, and stigmasterol for 8 (**a**) and 24 h (**b**), and HK2 protein expression was measured using flow cytometry. Data are presented as the mean ± SD (*n* = 3). ** *p* < 0.01.

Western blot images and relative fold changes in the TXNIP protein expression in MDA-MB-231 cells are presented in Figure 5a,b and Figure S2, respectively. TXNIP expression was significantly increased in cells treated with F3 (4-fold) and lutein (5-fold) compared to the untreated control (Figure 5b). The level of HK2 protein expression in MDA-MB-231 cells was determined by flow cytometry. Results show that the expression of HK2 was not affected by F3, lutein, β-sitosterol, and stigmasterol treatments (Figure 6). 

### 2.4. Effects of F3, Lutein, β-sitosterol, and Stigmasterol on Glucose Metabolism Regulatory Proteins

**Figure 7:**
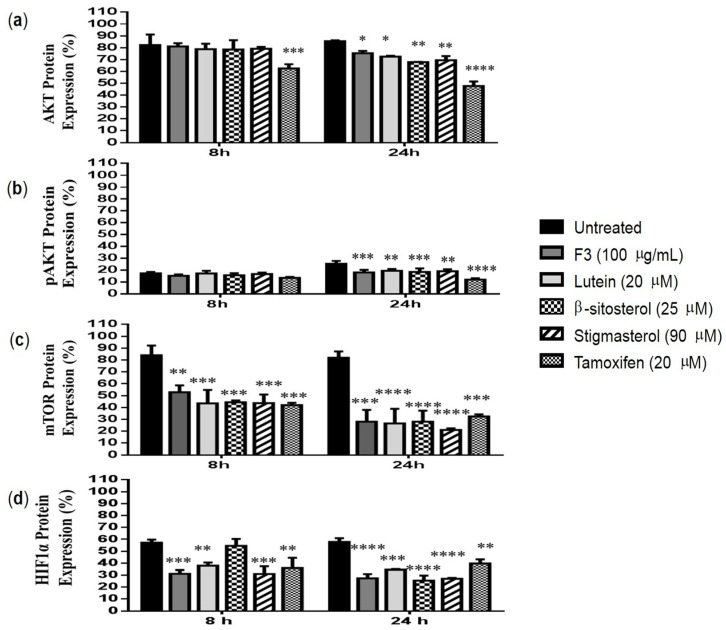
Expression of glycolysis regulatory protein. MDA-MB-231 cells were treated with IC_50_ concentrations of F3, lutein, β-sitosterol, and stigmasterol for 8 and 24 h. Protein expression of (**a**) AKT, (**b**) pAKT, (**c**) mTOR, and (**d**) HIF1α were measured using flow cytometry. Data are presented as the mean ± SD (*n* = 3). * *p* < 0.05; ** *p* < 0.01; *** *p* < 0.001; **** *p* < 0.0001.

Compared to the untreated control, a significant reduction in AKT expression (Figure 7a) was observed in MDA-MB-231 cells after being treated with F3, lutein, β-sitosterol, and stigmasterol for 24 h. Figure 7b further shows that the expression of phosphorylated AKT (pAKT) was significantly decreased compared to the untreated control, indicating the absence of the full activation of AKT. The protein expression of mTOR was also significantly reduced in MDA-MB-231 cells by F3 and its bioactive compounds. The reduction could be observed in as early as 8 h of treatments, with further reduction seen after 24 h (Figure 7c). Similarly, the expression of HIF1α was significantly reduced when MDA-MB-231 cells were treated for 24 h with F3, lutein, β-sitosterol, and stigmasterol, compared to the untreated control (Figure 7d). Our results also show that tamoxifen significantly inhibits the expression of AKT, pAKT, mTOR, and HIF1α proteins in MDA-MB-231 cells (Figure 7a–d).

### 2.5. Effects of F3, Lutein, β-sitosterol, and Stigmasterol on Metastatic-Related Activities

**Figure 8:**
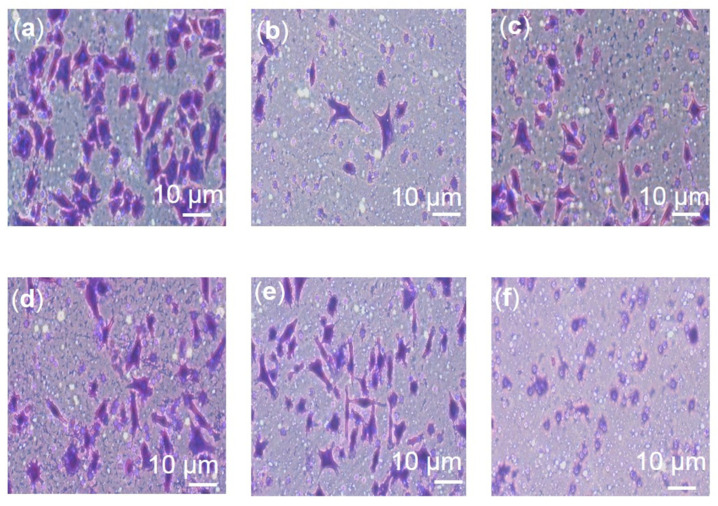
(**a**) Untreated; (**b**) F3 (100 µg/mL); (**c**) Lutein (20 µM); (**d**) β-sitosterol (25 µM); (**e**) Stigmasterol (90 µM); (**f**) Tamoxifen (20 µM). Determination of cancer cell migration by transwell migration assay observed under a microscope at 200× magnification.

**Figure 9:**
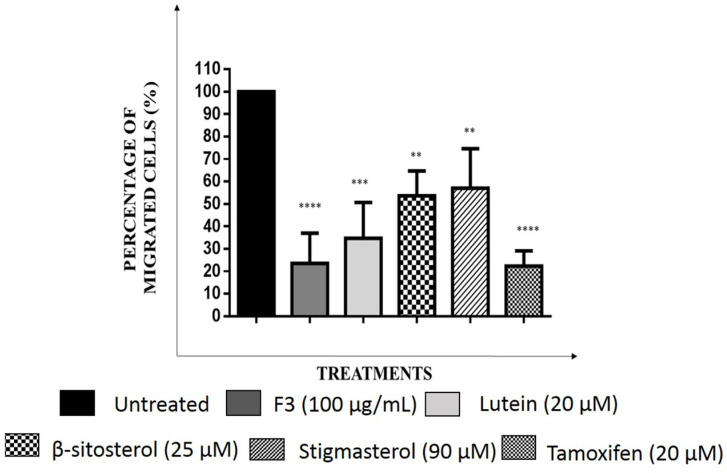
The percentage of migrated MDA-MB-231 cells, treated with IC_50_ concentrations of F3, lutein, β-sitosterol, and stigmasterol for 24 h, compared to control (untreated). Data are presented as the mean ± SD (*n* = 3). ** *p* < 0.01; *** *p* < 0.001; **** *p* < 0.0001.

**Figure 10:**
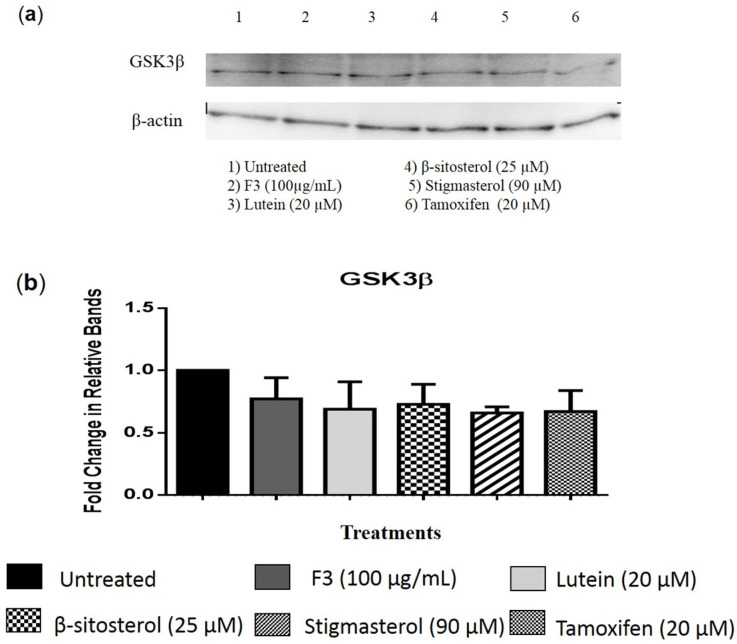
GSK3β protein expression. (**a**) Representative image of Western blot analysis of MDA-MB-231 cells treated with the indicated concentrations of (2) F3, (3) lutein, (4) β-sitosterol, (5) stigmasterol, and (6) tamoxifen (positive control), while (1) untreated as a negative control for 24 h. (**b**) Results are presented as an average fold change in the level of each protein normalized to β-actin ± SD (*n* = 3).

**Figure 11:**
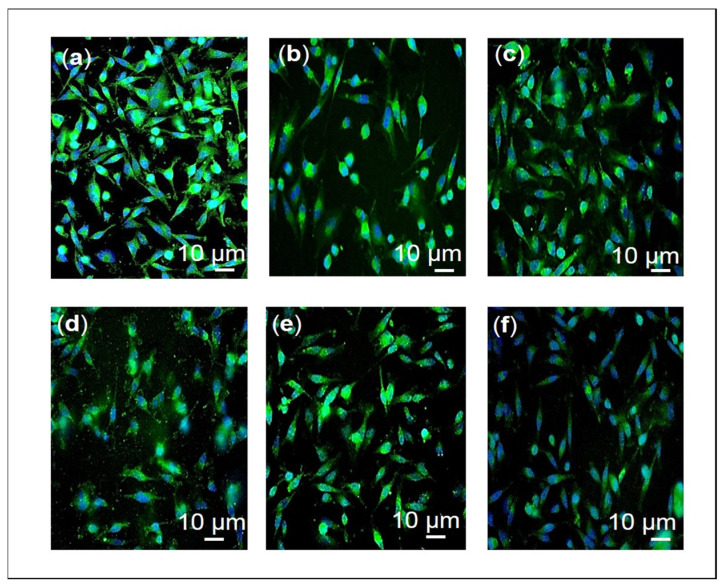
Fibronectin protein expression. The representative fluorescence image of MDA-MB-231 cells treated with the indicated concentrations (**b**) F3, (**c**) lutein, (**d**) β-sitosterol, and (**e**) stigmasterol for 24 h was compared to (**a**) control (untreated cells). Tamoxifen (**f**) was used as a positive control. Cells were observed at 400× magnification.

**Figure 12:**
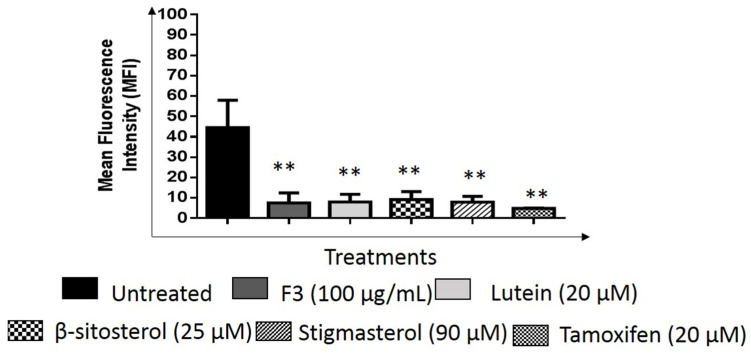
Fibronectin protein expression. MDA-MB-231 cells treated with the indicated concentrations F3, lutein, β-sitosterol, and stigmasterol for 24 h was compared to untreated cells. Tamoxifen was used as a positive control. Data are presented as the mean ± SD (*n* = 3). ** *p* < 0.01.

The relative number of migrated cells in the Transwell migration assay were quantified using the ImageJ software. As seen in Figure 8 and Figure 9, F3 and its bioactive compounds as well as tamoxifen significantly decreased the migration abilities of MDA-MB-231 after 24 h of treatment. GSK3β is known to mediate invasive phenotypes in cancer cells [34], therefore, GSK3β expression was then determined in the cells using Western immunoblotting as shown in Figure 10 and Figure S3. F3 and its bioactive compounds have no effects on the expression of GSK3β in the MDA-MB-231 cells, however. On the other hand, the expression of fibronectin, an EMT marker [35], was reduced after the treatment of MDA-MB-231 cells with F3, lutein, β-sitosterol, stigmasterol, and tamoxifen (Figure 11). This is evident by the reduction in the intensity of green fluorescence observed in the treated cells compared to the control (untreated cells) that showed high green intensity. Meanwhile Figure 12, showed significant reduction in mean fluorescence intensity in all treated cells when compared to control. 

## 3. Discussion

Aggressive and highly metastatic types of cancer cells show a high expression level of GLUT1 and GLUT3 [36]. This is because these cancer cells have a high glucose metabolism rate and, thus, require an enormous amount of energy from the uptake of glucose through GLUTs [37]. MDA-MB-231 cells are highly invasive breast cancer cells that overexpress GLUT1 [38,39]. These cells have been shown to exhibit the characteristics of Warburg effects based on the correlation between the acidification of the external tumor microenvironment and lactic acid production [40]. Interestingly, in comparison to MCF-7 cells (non-metastatic breast cancer cell line), MDA-MB-231 cells internalize a significant amount of glucose [37]. Based on our previous findings, F3 inhibits glucose uptake in MDA-MB-231 cells [32]. The inhibition of glucose uptake by F3 was reported to be due to the presence of lutein and β-sitosterol as those two compounds were found to inhibit glucose uptake activity in the MDA-MB-231 cells. 

Inhibition of glucose transporters may become a potential therapeutic approach for cancer disease because it leads to the inhibition of glucose uptake into the cells [41]. There are several methods for the inhibition of glucose transporters such as the use of drug inhibitors, antibodies that specifically target the glucose transporter, and siRNA [41]. Examples of glucose transporter inhibitors that have been reported on cancer cells are STF31 (a small molecule that belongs to the second class in the group of pyridyl aniline thiazoles compounds) [42], WZB117 (resveratrol, a phytochemical present in various fruits including grapes) [43], phloretin (a natural phenol in apple leaves) [44], and many more. Changes in the individual transporter efficiency and the number of transporters at the plasma membrane can influence the uptake of glucose through GLUTs [45,46]. Other than the increased expression of GLUT1, the transportation of vesicles containing GLUT1 from the intracellular components into the cell membrane also increased when the cells were in hypoxic conditions [47]. Another major form of GLUT1 regulation is through the subcellular trafficking between the internal vesicular compartment and the cell membrane [48]. The enhancement of GLUT1′s membrane localization requires phosphorylation of GLUT1 at the S226 site, whereas GLUT1 cell surface trafficking can be disrupted by deletion of the PDZ binding motifs [49,50]. Furthermore, the internalization of GLUT1 can occur via the early endosomes (EE) and late endosomes (LE), which can then be transported into the lysosome, via the endolysosomal pathway, where it will be destroyed. However, GLUT1 can be recycled back to the cell membrane through the recycling endosome (RE) [51,52]. Thus, the trafficking activity of GLUT1 from the cytosol to the cell membrane may play a role in glucose uptake in cancer cells. In the current study, the inhibition of glucose uptake in MDA-MB-231 cells by F3 and its bioactive compounds (lutein and β-sitosterol) was not accompanied with a downregulation in the expression of GLUT1. However, fluorescence microscopy analysis showed that localization of GLUT1 to the cell membrane was inhibited by F3, lutein, and β-sitosterol. Interestingly, our current study also shows that stigmasterol did not inhibit localization of GLUT1 to the cell membrane, which supported the lack of inhibition of glucose uptake by stigmasterol reported previously [32].

For over 30 years, PKC activation has been known to rapidly initiate glucose uptake by phosphorylating GLUT1 at serine 226 [50,53]. This S226 is located in a conserved sequence in the large intracellular loop, together with the C-terminal tail of GLUT1, to regulate its trafficking and activity [54]. By using phorbol esters to activate PKC, Lee and his colleagues [50] demonstrated that this activation caused an increase in the phosphorylation of GLUT1 S226 and later increased GLUT1 localization to the cell surface. Hence, decreased PKC activity observed in the current study provides support for the inhibition of GLUT1 localization to the MDA-MB-231 cell membrane by F3, lutein, and β-sitosterol. 

TXNIP is another regulator for GLUT1 plasma membrane localization [55]. TXNIP expression is often downregulated in cancer cells and the suppression of TXNIP expression in normal cells increases the risk of cancer [56]. In our study, TXNIP protein expression increased when MDA-MB-231 cells were treated with F3, lutein, β-sitosterol, or stigmasterol. An increased TXNIP protein expression has been reported to negatively regulate glucose metabolism in the cells by binding to GLUT1 and facilitating its endocytosis via clathrin-coated pits (CCP) [21]. This further supports our finding that the inhibition of glucose uptake in the TNBC cells by F3, lutein, and β-sitosterol is attributed to the inhibition of GLUT1 plasma membrane localization. 

An increased level of TXNIP expression is correlated with the inhibition of the P13K/AKT/mTOR pathway [57,58]. The P13K/AKT signaling pathway is known for its role in tumorigenesis and it can activate many downstream proteins involved in the proliferation, survival, and apoptosis of cancer cells [59]. Furthermore, it is also involved in the reprogramming of cancer cell metabolism [60]. Increased expression of AKT activity will increase the rate of glycolysis in the cancer cells. This is mainly due to the function of activated AKT in regulating the localization of the GLUT1 to the plasma membrane [61,62,63] as well as regulating hexokinase expression and its activity [64]. In order for the AKT to be fully activated, phosphorylation at S473 and T308 sites by mTORC2 and PDK1, respectively, is required [65,66,67]. We show that treatments with F3 or its bioactive compounds significantly decreased the expression of both AKT and pAKT (at the S473 site) at 24 h, as compared to the untreated control. 

During glycolysis, glucose is phosphorylated to glucose-6-phosphate by HK2. Recent reports showed that blocking the PI3K/AKT signaling pathway could suppress HK2 expression in both hepatocellular carcinoma and breast cancer cells [68,69]. AKT activation promotes HK2 association with the outer mitochondria membrane and interaction with the voltage-dependent anion channel (VDAC) for immediate access to the ATP [70,71]. However, when the level of glucose-6-phosphate increases, HK2 will be translocated into the nucleus, which leads to the inhibition of HK2 activity as well as autophagic effects [72,73]. The translocation of HK2 to the nucleus also occurs when the level of AKT expression in the cells decreases due to VDAC phosphorylation. This causes dissociation of HK2 from the mitochondria [74], which promotes apoptosis in cancer cells [75]. In this study, F3 and its bioactive compounds did not inhibit the expression of HK2 in the MDA-MB-231 cells. Nevertheless, the reduced expression of pAKT could lead to an inhibition of HK2 activity. 

The mammalian target of rapamycin complex (mTORC) is a downstream target for the PI3K/AKT pathway. mTOR is known as a master regulator in diverse biological processes such as cell proliferation, survival, autophagy, and metabolism [76,77]. Hong and his colleagues [57] reported that constitutive activation of PI3K/AKT/mTOR signaling leads to TXNIP downregulation. Downregulation of mTOR protein expression in the MDA-MB-231 cells was observed after treatment with F3 and its bioactive compounds, which, hence, leads to the increased expression of TXNIP in the treated cells. Another regulator for aerobic glycolysis in cancer cells is HIF1α, and this protein is positively enhanced by the activation of mTOR [78]. The expression of HIF1α can induce the expression of genes that encode glycolytic enzymes and regulate the uptake of glucose through GLUTs [79,80,81]. In the current study, downregulation of mTOR by F3 and its bioactive compounds also caused a reduction in the expression of HIF1α. The downregulation of HIF1α expression in cells treated with F3, lutein, β-sitosterol, and stigmasterol can explain the ability of these compounds to reduce lactate concentration in the MDA-MB-231 cells [32] as HIF1α can regulate the expression of LDHA [82]. Although HIF1α expression is not associated with the total GLUT1 expression, the membrane level of GLUT1 seems to be regulated by HIF1α as reported in thyroid cancer cells [83]. 

Lactate that is produced by cancer cells during the Warburg effect will be exported into the extracellular space via monocarboxylic transportation (MCT) and generates microenvironmental acidosis with a pH lower than 6.0 [69,84]. This condition has been demonstrated to modulate the expression of epithelial mesenchymal transition (EMT) related proteins as well as affect the stability of the tissue structure [85]. Furthermore, the dysregulation of pH in cancer cells is described to promote the proliferation, metastasis, and evasion of apoptosis [86]. Based on our Transwell migration assay, F3 and its bioactive compounds were shown to reduce the percentage of migrated cells. The results are concomitant with other findings that proved lutein inhibits migration of breast cancer cells and reduces the expression of EMT-related proteins [87]. β-sitosterol reduces invasion of MDA-MB-231 cells, and protects against metastasis [88], and stigmasterol exerts anti-migratory effects on ovarian and gastric cancer cells [85,86,89,90].

GSK3β, a serine/threonine protein kinase, can act as a tumor suppressor or tumor promoter depending on the types of cancer cells [91]. In breast cancer cells, overexpression of GSK3β indicates poor prognosis and increases the risk of relapse [92]. Knockdown of GSK3β in breast cancer cells has been shown to inhibit cellular proliferation [93]. MDA-MB-231 cells are TNBC cells that have undergone EMT and are in dominance with the mesenchymal cell attributes [94]. Based on a study by Vijay and colleagues [95], GSK3β inhibitors can inhibit the EMT and migratory properties of TNBC cells. However, our findings show that F3 and its bioactive compounds have no effects on the expression of GSK3β in the MDA-MB-231 cells, although anti-migratory activity was observed. Besides showing morphological changes, cancer cells undergoing EMT also exhibit molecular alterations as demonstrated by the decreased expression of epithelial markers (for example, E-cadherin, ZO-1, and occludin) and increased expression of mesenchymal markers (for example, N-cadherin, vimentin, fibronectin, and fibroblast-specific protein) [96,97,98]. Previously, we demonstrated that F3 had anti-metastatic activity in breast cancer by reducing the expression of N-cadherin, vimentin, MMP-9, MUC1, Twist, and VEGF, while increasing the expression of E-cadherin [28]. In the current study, the expression of fibronectin was also reduced. Fibronectin is a component and regulator of ECM that plays a role in cancer progression such as migration, invasion, and metastasis [99,100,101]. Consequently, silencing fibronectin expression in renal cancer cells was reported to decrease cell migration [102]. Similarly, F3, lutein, β-sitosterol, and stigmasterol were found to inhibit the migration of MDA-MB-231 cells and reduce the expression of fibronectin. Fibronectin was also reported to increase the migration and invasion of ovarian cancer cells through the activation of focal adhesion kinase (FAK) and the increase of the signaling regulation of FAK, which is the PI3K/AKT pathway [103]. Cell migration involves assembly and disassembly of focal adhesion complexes, adhesion sites between cells, and ECM [104]. Hence, FAK becomes an important regulator for cell migration as it controls the turnover of the adhesion sites [105].

## 4. Materials and Methods

### 4.1. Cell Culture and Treatments

The MDA-MB-231 cell line was purchased from the American Type Culture Collection (ATCC, Manassas, VA, USA) and cultured in Dulbecco’s modified Eagle’s medium (DMEM) (Nacalai Tesque, Kyoto, Japan). The media was supplemented with 10% fetal bovine serum (FBS) (Thermo Fisher Scientific, Shanghai, China), 100 IU/mL penicillin, and 100 µg/mL streptomycin (Thermo Fisher Scientific, Shanghai, China).

The cells were treated with 100 µg/mL F3, which was sourced from the Universiti Sains Malaysia Centre for Drug Research. The F3 was prepared from pulverized freeze-dried leaves of *S. crispus* as previously reported [29,30]. MDA-MB-231 cells were also treated with 20 µM lutein, 25 µM β-sitosterol, or 90 µM stigmasterol (Cayman, MI, USA), based on the IC_50_ concentrations obtained from the MTT assay [32]. Apigenin was used as a positive control for the detection of GLUT1 because it has been shown to inhibit both GLUT1 mRNA and protein expression [106,107]. Meanwhile, tamoxifen was used as a positive control in all subsequent experiments because it has previously been reported to decrease PKC activity and affect the PI3K/AKT pathway in MDA-MB-231 cells [108,109].

### 4.2. Immunofluorescence Analysis

MDA-MB-231 cells were grown on a chamber slide (Thermo Fisher Scientific, Waltham, MA, USA) overnight, prior to 24 h treatments with F3, lutein, β-sitosterol, or stigmasterol. The cells were then washed with phosphate-buffered saline (PBS) and fixed with 4% paraformaldehyde (in PBS) at room temperature (RT) for 10 min. In order to permeabilize and block the non-specific protein–protein interactions, cells were incubated for 1h with 1% BSA in 0.1% PBS-Tween 20. Cells were then washed once with PBS, followed by incubation with either the anti-glucose transporter GLUT1 (ab652) (Abcam, Cambridge, UK) (1:1000), or the fibronectin antibody (960632) (Novus Biologicals, Englewood, CO, USA) (1:1000), in a blocking reagent overnight at 4 °C. Then, the cells were washed with PBS and incubated with the secondary antibodies of Alexa Fluor and the Goat Anti-Rabbit IgG (Abcam, Cambridge, UK) for 1h in the dark at RT. The cells were then washed with PBS and the coverslips were mounted with Dako Fluorescent Mounting Medium containing DAPI (Agilent Technologies, Santa Clara, CA, USA) prior to microscopy. Images were acquired on 40× objective using an OLYMPUS BX41 Fluorescent microscope (Olympus, Tokyo, Japan). Representative images are shown in the figures at the same exposure, magnification, and in merged color images.

### 4.3. Cell Lysis and Western Blot Analysis

Cells were lysed with 10× RIPA buffer (Cell Signaling Technology, Danvers, MA, USA) and washed with PBS, before adding 1× RIPA buffer to the plate, and incubated on ice for 5 min. The cells were collected and centrifuged at 14,000× *g* for 10 min. A total of 20 µg of protein lysates were resolved in SDS-PAGE. Membranes were probed overnight with primary antibodies of GLUT1 (1:1000) (Abcam, Cambridge, UK), TXNIP Recombinant Rabbit mAb (1:500) (Thermo Fisher Scientific, Waltham, MA, USA), GSK-3β antibody (1:500) (Novus Biological, Englewood, CO, USA), and β-actin Rabbit mAb (Thermo Fisher Scientific, Waltham, MA, USA) (1:1000). The proteins were detected using chemiluminescence (Alpha Innotec, Kasendorf, Bayern, Germany) after incubation with a secondary antibody of Goat Anti-Rabbit IgG H&L HRP (Abcam, Cambridge, UK) for 1 h.

### 4.4. Flow Cytometric Analysis

MDA-MB-231 cells were seeded on a 6-well plate and treated with F3, lutein, β-sitosterol, or stigmasterol for 8 and 24 h. Cells were harvested and washed with PBS. Cell Fixation and Permeabilization Kits (Abcam, Cambridge, UK) were used for intracellular protein detection. The harvested cells were fixed with a fixation medium (Reagent A) for 15 min, washed with PBS, followed by a permeabilization medium (Reagent B) for 15 min. Next, the cells were washed with PBS and stained with primary antibodies that had been diluted in 3% BSA/PBS for 2 h. The primary antibodies were AKT (pan) Rabbit mAb (Cell Signaling Technology, Danvers, MA, USA), phospho-AKT Rabbit mAb (Cell Signaling Technology, Danvers, MA, USA), mTOR Rabbit mAb (Cell Signaling Technology, Danvers, MA, USA), and HIF1α polyclonal (Thermo Fisher Scientific, Waltham, MA, USA). The incubated cells were then washed with PBS and the Goat Anti-Rabbit IgG H&L (Alexa Fluor) (Abcam, Cambridge, UK) secondary antibody (diluted in 3% BSA/PBS) was added to the cells for 1h. Lastly, the cells were washed with PBS and analyzed using flow cytometry with 10,000 cells per sample detected.

### 4.5. PKC Activity Analysis

Protein kinase C activity in treated MDA-MB-231 cells was measured using the ADI-EKS-420A assay kit (Enzo Life Sciences, Farmingdale, NY, USA). Cells were first lysed using 10× RIPA buffer (Cell Signaling Technology, Danvers, MA, USA). Following the manufacturer’s protocol, 30 µL of lysate per sample was used to measure the PKC activity at a 450 nm wavelength using a spectrophotometer (Molecular Devices, San Jose, CA, USA). The result was expressed as a relative activity of PKC.

### 4.6. Migration Assay

Transwell migration assay was performed by seeding MDA-MB-231 cells (5 × 10^5^ cells/well) in the cell culture insert (8.0 µm pore size) (SPL Life Sciences, Gyeonggi-do, Korea) containing 1% FBS with or without treatment. To create a chemoattractant gradient to the cells, 10% FBS (with or without treatment) was added to the bottom of the Transwell system. After 24 h, cells that migrated through the membrane in response to chemoattractant were then fixed with methanol for 10 min followed by staining with 0.05% crystal violet solution for 15 min. The excess staining solution was rinsed with distilled water. The results are reported as average numbers of stained cells counted in ten random fields (200× magnification) under a light microscope.

### 4.7. Statistical Analysis

Statistical significance was determined using either One-Way ANOVA or Two-Way ANOVA analyzed with the GraphPad Prism version 6.01 (Boston, MA, USA) software. Results were obtained from three independent experiments and presented as means ± standard deviations (SD).

## 5. Conclusions

The *S. crispus* subfraction F3 exerts its anti-cancer effects in breast cancer cells by modulating the glycolytic and migratory activities via the inhibition of GLUT1 localization to the cell membrane and the inhibition of the AKT/mTOR/HIF1α pathway that potentially inhibits the expression of glycolytic-related molecules. Metabolic changes exerted by F3 modulate the expression of EMT-related proteins in the breast cancer cells. These anti-cancer effects of F3 are attributed to the presence of its bioactive compounds such as lutein, β-sitosterol, and stigmasterol. Since metabolic rewiring can induce cancer metastasis and drug resistance, further studies are then required to know the effects of those compounds on the EMT-induced drug resistance in MDA-MB-231 cells.

## Data Availability

Not applicable.

## Supplementary Materials

The following supporting information can be downloaded at: https://www.mdpi.com/article/10.3390/ph16020153/s1, Figure S1. Representative image of (a) GLUT1 and (b) β-actin protein expression in MDA-MB-231 cells treated with the indicated concentrations of (2) F3, (3) lutein, (4) β-sitosterol, (5) stigmasterol, and (6) apigenin (positive control), while (1) untreated as a negative control for 24 h; Figure S2. Representative image of TXNIP protein expression in MDA-MB-231 cells treated with the indicated concentrations of (2) F3, (3) lutein, (4) β-sitosterol, and (5) stigmasterol for 24 h. Tamoxifen (6) was used as a positive control, while untreated cells (1) as a negative control; Figure S3. Representative image of (a) GSK3β and (b) β-actin protein expression in MDA-MB-231 cells treated with the indicated concentrations of (2) F3, (3) lutein, (4) β-sitosterol, (5) stigmasterol, and (6) tamoxifen (positive control), while (1) untreated as a negative control for 24 h.

## Abbreviations

GLUT	glucose transporter
TXNIP	Thioredoxin Interacting Protein
PKC	protein kinase c
AKT	Ak strain transforming
pAKT	phosphorylated Ak strain transforming
mTOR	mammalian target of rapamycin
HIF1α	Hypoxia-inducible factor 1-alpha
HK2	hexokinase 2
PKM2	pyruvate kinase type M2
LDH	lactate dehydrogenase
MCT	monocarboxylate transporters
ROS	reactive oxygen species
EMT	epithelial-mesenchymal transitions
ECM	extracellular matrix
CTC	circulating tumor cells
TNBC	triple negative breast cancer cells
NMU	N-Methyl-N-nitrosourea
GSK3β	Glycogen synthase kinase-3 beta
PDK1	Pyruvate dehydrogenase kinase 1
VDAC	voltage-dependent anion channel
