# Supplementary material for: Regulatory Mechanism on Anti-Glycolytic and Anti-Metastatic Activities Induced by Strobilanthes crispus in Breast Cancer, In Vitro
Source: Pharmaceuticals (Basel). 2023 Jan 20;16(2):153. doi: 10.3390/ph16020153 (PMC9963282; doi:10.3390/ph16020153)
Supplement: Supplementary file 1 [file pharmaceuticals-16-00153-s001.zip › pharmaceuticals-2031777-supplementary.pdf]

(a)

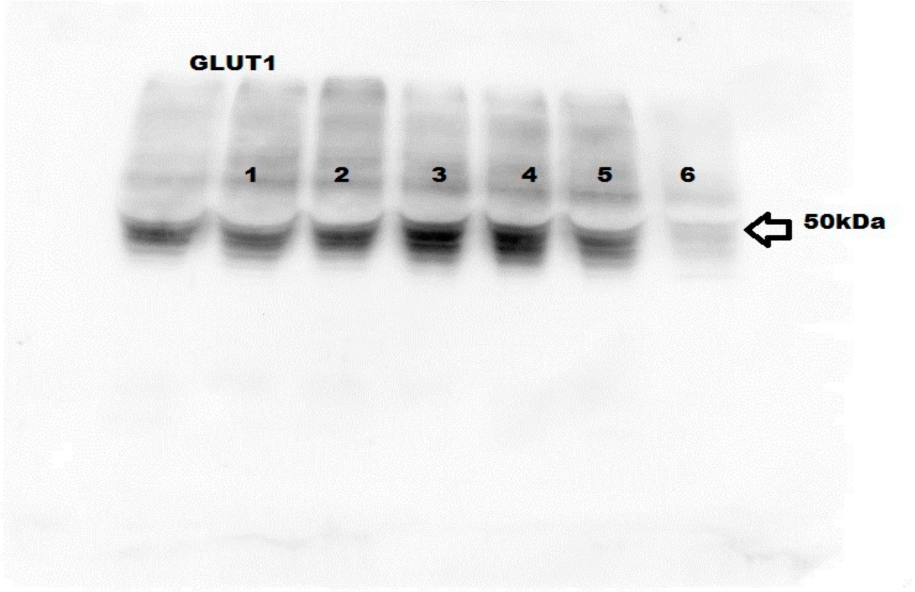

(b)

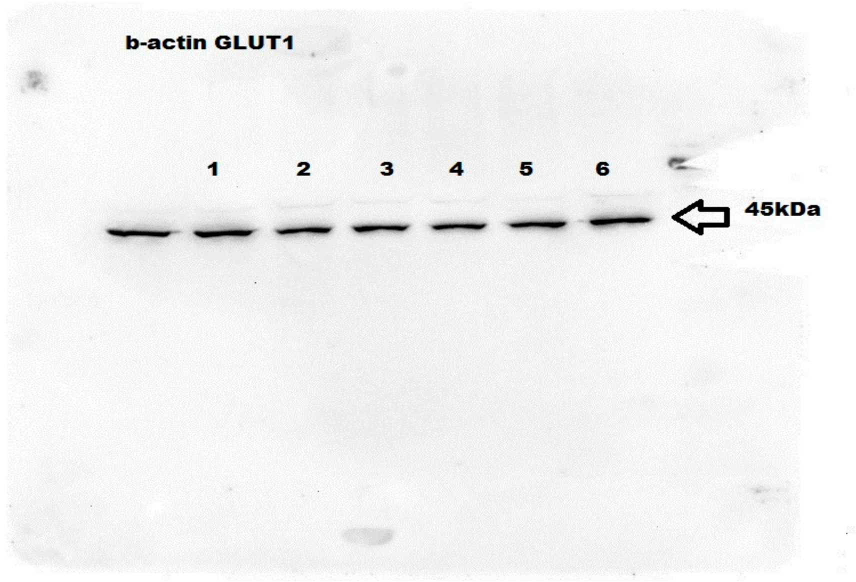

Figure S1. Representative image of (**a**) GLUT1 and (**b**) β -actin protein expression in MDA-MB-231 cells treated with the indicated concentrations of (2) F3, (3) lutein, (4) β-sitosterol, (5) stigmasterol, and (6) apigenin (positive control), while (1) untreated as a negative control for 24 h.

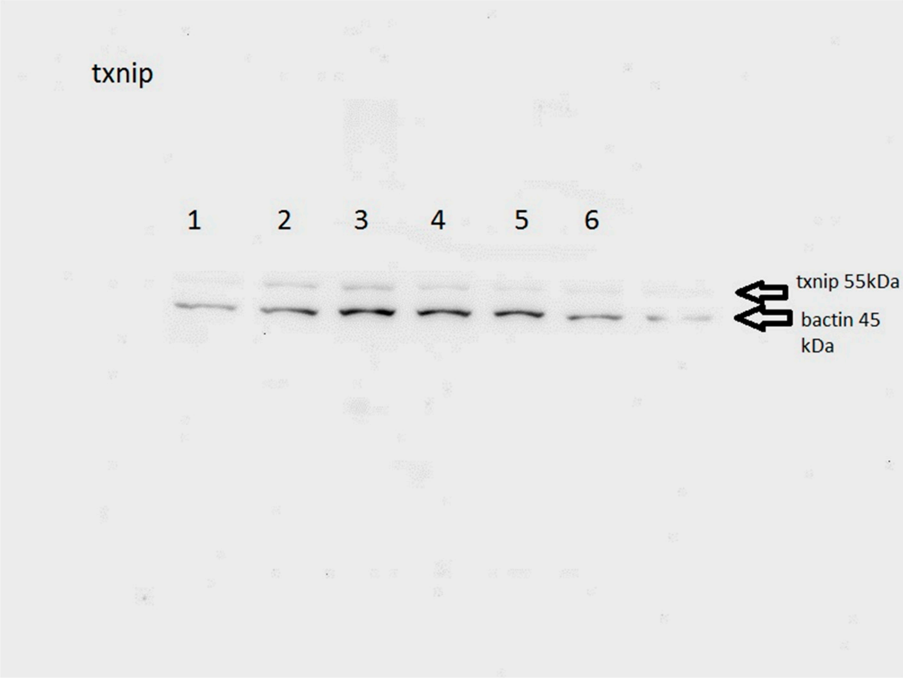

Figure S2. Representative image of TXNIP protein expression in MDA-MB-231 cells treated with the indicated concentrations of (2) F3, (3) lutein, (4) β-sitosterol, and (5) stigmasterol for 24 h. Tamoxifen (6) was used as a positive control, while untreated cells (1) as a negative control.

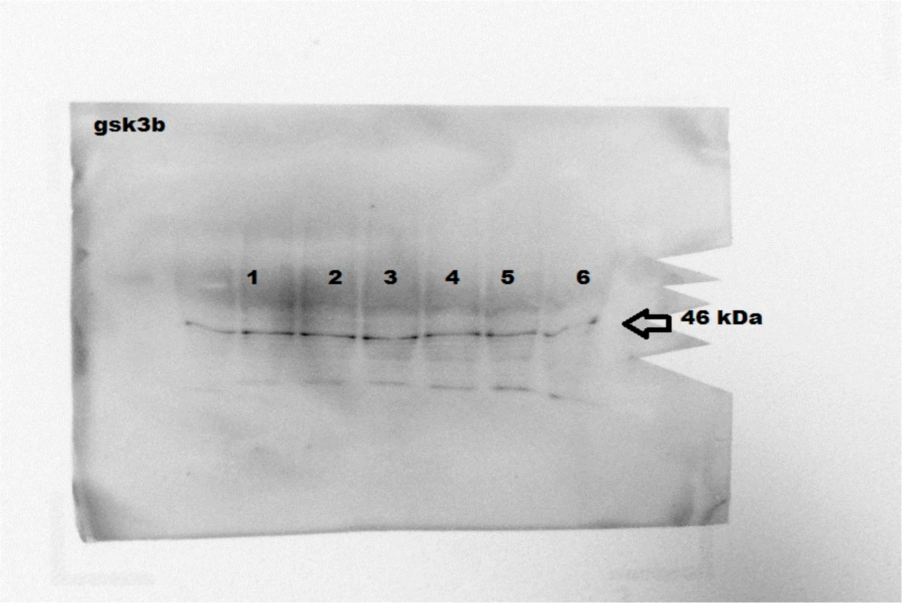

(**a**)

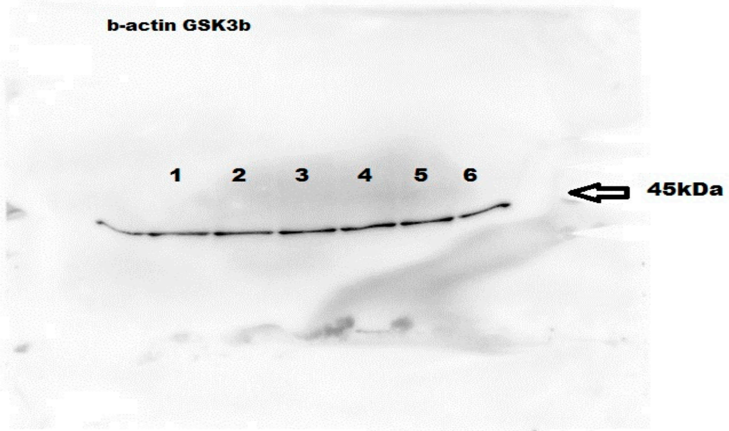

(**b**)

Figure S3. Representative image of (**a**) GSK3β and (**b**) β-actin protein expression in MDA-MB-231 cells treated with the indicated concentrations of (2) F3, (3) lutein, (4) β-sitosterol, (5) stigmasterol, and (6) tamoxifen (positive control), while (1) untreated as a negative control for 24 h.
